# Electrolysis

**Published:** 1877-07-20

**Authors:** 


ELECTROLYSIS.

Dr. Cutler, of Massachusetts, at the American Medical Association, exhibited an apparatus devised by him for the electrolysis of uterine tumors. Pointed electrodes are driven into the tumor, sometimes through the abdominal wall, or through rectum, or vagina. The electrodes must be separated from each other by at least a half inch. Ether should be employed during the operation, and the sitting may be from five to ten minutes.

